# Platelet indices and inflammatory bowel disease: a Mendelian randomization study

**DOI:** 10.3389/fimmu.2024.1377915

**Published:** 2024-07-09

**Authors:** Hong-yang Li, Tie-mei Liu

**Affiliations:** Department of Blood Transfusion, China-Japan Union Hospital of Jilin University, Changchun, China

**Keywords:** Mendelian randomization, platelet indices, inflammatory bowel disease, Crohn’s disease, ulcerative colitis

## Abstract

**Background:**

Platelets play a significant role in the innate and adaptive processes of immunity and inflammation. Inflammatory bowel disease (IBD) is an autoimmune disease that is widely understood to be caused by a combination of genetic predisposition, aberrant immune responses, etc.

**Methods:**

To examine the relationships between genetically determined platelet indices and IBD, we conducted a Mendelian randomization (MR) study. Data associated with platelet count (PLT), mean platelet volume (MPV), platelet distribution width (PDW), plateletcrit (PCT) were used from the UK Biobank. The outcome data, including IBD, Crohn’s disease (CD), ulcerative colitis (UC), were from the FinnGen database. The inverse variance-weighted (IVW), MR-Egger, weighted median methods were used for MR analyses.

**Results:**

The MR estimations from the IVW approach show a significant connection between PLT and IBD. Similarly, PCT and IBD have a relationship following the IVW and MR-Egger approaches. While PLT and PCT have strong relationships with CD, according to the findings of all three approaches respectively. Nevertheless, PDW was the only relevant indicator of UC. The only significant result was IVW’s.

**Conclusion:**

Our findings suggest that the fluctuation of platelet indicators is of great significance in the development of IBD. PLT and PCT have a close association with IBD and CD, respectively; PDW only has a connection with UC. Platelets play an important role in the progression of IBD (UC, CD).

## Introduction

Platelets are blood cells in plasma that are well recognized for their critical role in sustaining blood hemostasis (1). Megakaryocytes (MKs) create billions of them every day. MKs perceive and respond to inflammatory stress, and they engage in host immunological responses, according to emerging data (2). Platelet count (PLT), mean platelet volume (MPV), platelet width of distribution (PDW), and plateletcrit (PCT) are major platelet indicators in clinical practice that may be utilized to indicate platelet biochemical and functional changes (3). Platelets also play important roles in innate and adaptive immunity and inflammation, and they are the first blood cells to respond to wound-healing and tissue-repair mechanisms (1). Small platelets manage to maintain vascular integrity when faced with challenges of infection, sterile inflammation, and even malignancy, where they aid in hemostasis and serve as early responders to microbial threats (4). Because of their quick recruitment dynamics, these tiny, anucleate cell fragments are the first cells to form not just at sites of damage but also at sites of inflammation (5). Intravital imaging indicated that platelets are recruited and behave as individual cells rather than clots in the inflamed microvasculature, indicating that the hemostatic mechanism is unique to classical thrombosis and hemostasis. Unlike the well-defined processes of hemostasis following vascular trauma, inflammation-associated hemorrhage, also known as inflammatory bleeding, is a simplified summary of a phenomenon that occurs in a variety of disease settings, including sterile inflammation, microbial infection, and malignant tumors (6–8). Predilection sites include mucosal membranes, with epistaxis, gum bleeding, gastrointestinal bleeds, and hematuria being the most common bleeding episodes in thrombocytopenia patients. Platelet-mediated hemostasis without clot formation is critical to maintaining vascular integrity under these conditions (9, 10).

The autoimmune illness known as inflammatory bowel disease (IBD) is a chronic, relapsing condition that has caused significant health problems and is becoming more commonplace worldwide (11, 12). It is well accepted that genetic predisposition, environmental variables, and abnormal immune responses combine to cause IBD (13). The two main IBD subtypes, ulcerative colitis (UC) and Crohn’s disease (CD), can differ significantly in terms of their molecular, immunological, morphological, and clinical features (14). Rectal bleeding, diarrhea, stomach discomfort, fever, anemia, and weight loss are some of the symptoms of UC (15, 16). CD may impact any region of the digestive tract in addition to causing diarrhea and abdominal pain (17). Up to 29.3 percent of IBD patients have at least one extra intestinal manifestation (EIM), which can have an effect on many systems, according to a Swiss cohort study (18). As per the European Crohn’s and Colitis Organization, at least one EIM is experienced by up to 50% of people with IBD (19). Because of its great prevalence, IBD not only drastically lowers patients’ quality of life but also places a major financial and medical burden on society (20), additionally accompanied by a number of issues or EIM (21). The most common areas of the body affected by the various types of EIMs are the musculoskeletal system, mucocutaneous system, ocular system, hepatobiliary tract, and oral cavity. There’s a chance that other systems, including the pancreatic, pulmonary, cardiovascular, and urogenital systems are also at play (22, 23). Hematological EIMs haven’t been thoroughly acknowledged or verified yet. Although the exact pathogenesis of EIMs is still unknown, it often involves dysregulated immunological responses, environmental factors, genetic vulnerability, and microbiota dysbiosis (19). Therefore, in order to obtain better prevention and control, it is essential to investigate the pathophysiology and risk factors of IBD. Determining causative relationships and possible risk factors for IBD represents an emerging public health concern.

A recent research by Vallet et al., which was published in the Journal of Clinical Investigation (24), demonstrates how the locations of megakaryocytes and the quality of platelet production alter with illness. Considering the vital role platelets play in coagulation, wound healing, tissue damage repair, immunological response, and inflammatory infections. Thus, assessments of platelet indices that reflect platelet bioactivity may be extremely important for tracking the onset, course, management, and prognosis of IBD.

In conclusion, it has not been established that platelet indices and IBD (UC and CD) are causally related. However, conventional observational study designs are limited in their ability to establish causality regarding the function of platelets in the development of IBD because of significant methodological constraints like reverse causation and residual confounding. A different strategy is the Mendelian randomization (MR) design, which makes use of genetic variations as instrumental variables (IVs) for an exposure in order to establish the causal relationship between the exposure and the outcome (25–28). By employing genetic variation as an indicator of causation, MR can remove the confounding bias seen in observational research. As alleles follow the principle of random assignment, different genotypes result in different intermediate phenotypes. If this phenotype represents an individual’s exposure characteristic, the association effect between genotype and disease can describe the impact of exposure factors on illness. This effect is unaffected by confounding factors and reverse causal associations, as in traditional epidemiological studies (25, 29). The MR study concept is founded on Mendel’s rule and functions similarly to a randomized controlled trial (RCT) but without the high expense (30).

In the current investigation, we employed a two-sample MR analysis to ascertain the association between platelet indices (PLT, MPV, PDW, and PCT) and IBD (UC and CD). It suggests that an IV-induced modifiable exposure caused the result. Therefore, we think the single-nucleotide polymorphisms (SNPs) used as research instruments had a modifying impact on the platelet indices, proving a positive causal relationship between the SNPs and the probability of developing IBD. However, interventions aimed at targeting the exposure are unlikely to be effective if there is a non-causal link between the exposure and the outcome.

## Materials and methods

### Study design

**Figure 1:**
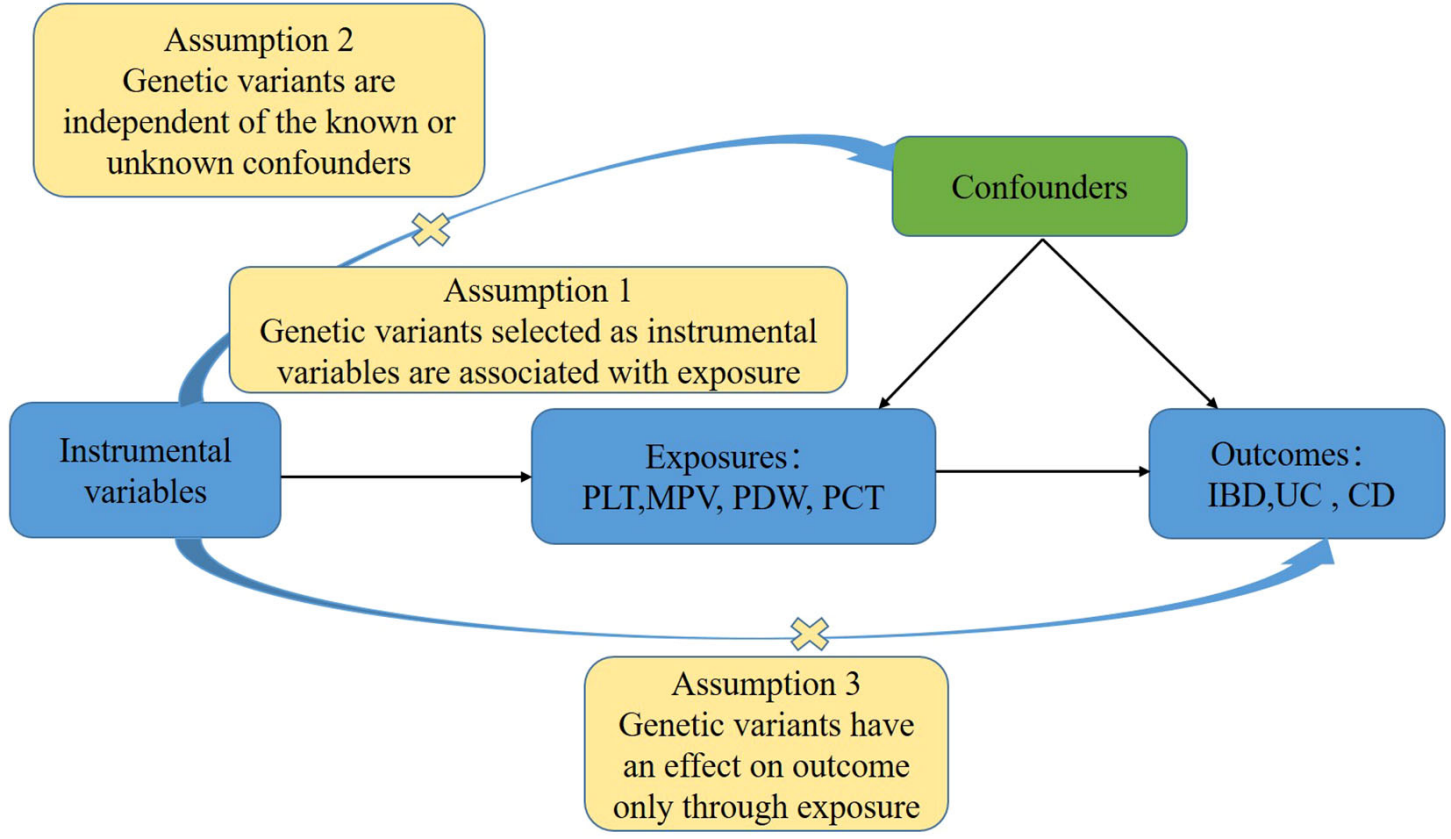
The basic principles of the MR study show the three principal assumptions.

**Figure 2:**
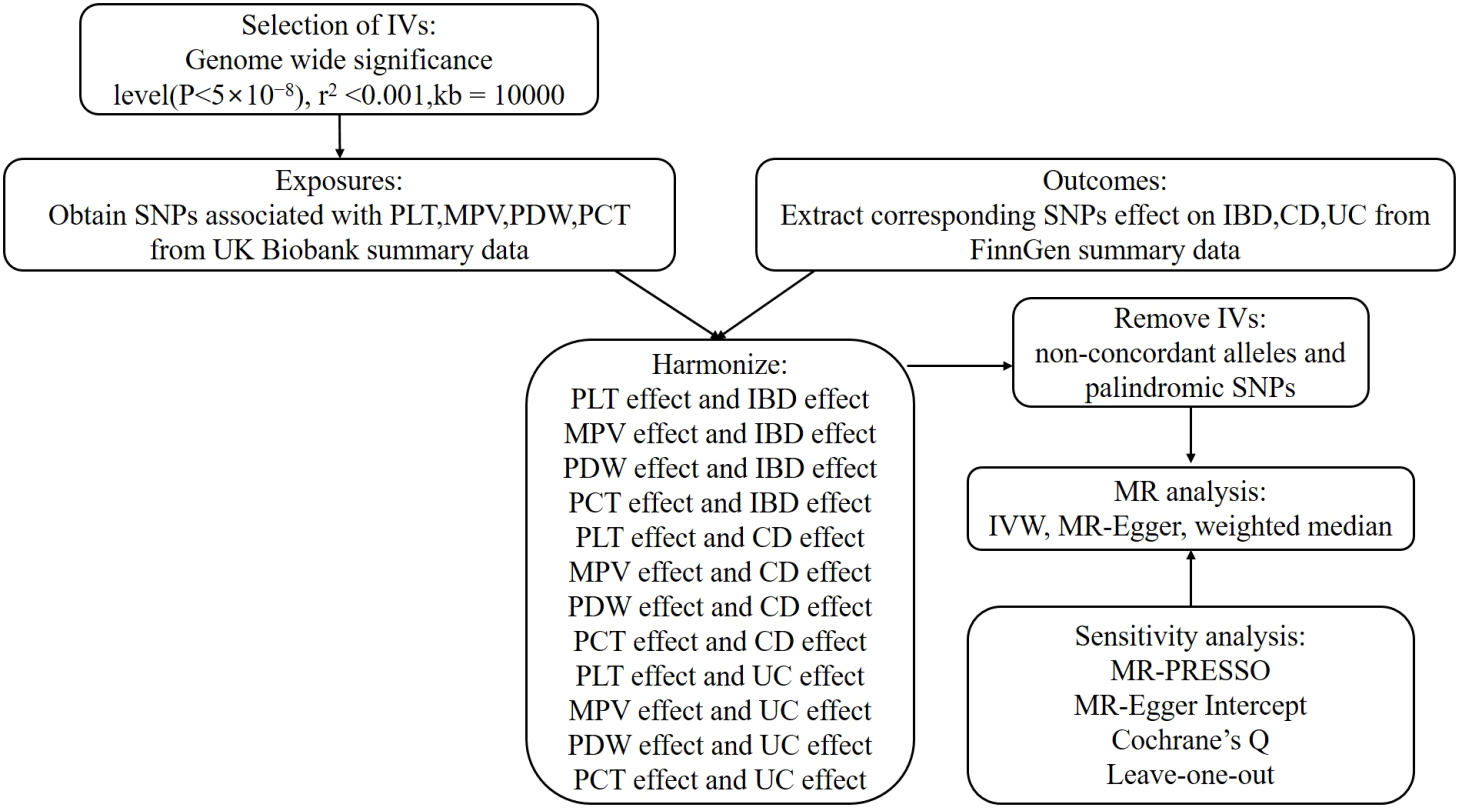
An overview of the study design.

In order to investigate the associations between platelet indices (PLT, MPV, PDW, and PCT) and IBD (UC and CD), we used a two-sample MR design. Strengthening the Reporting of Observational Studies in Epidemiology Using Mendelian Randomization and the Fundamentals of MR were adhered to in the design of our study (31). Additionally, these selections underwent an MR analysis and satisfied three fundamental presumptions (**Figure 1**): Three things are relevant about the instrumental variables: (1) they are directly correlated with the exposure; (2) they are unaffected by confounders; and (3) genetic variations only influence outcomes through exposure (32). The purpose of the univariable MR study was to explore the relationship between platelet indices (PLT, MPV, PDW, and PCT) and IBD (UC and CD). The research design employed is shown in **Figure 2**.

### Data source

The genetic tools for the four platelet indices (PLT, MPV, PDW, and PCT) were chosen from a genome-wide association study (GWAS) that involved 408,112 participants in the UK Biobank (33). Every participant was descended from Europeans. Data from the FinnGen collaboration, which became publicly available in May 2021, was utilized to determine the outcomes. Which enrolled 218,792 European participants (cases/controls for IBD: 5,673/213,119; CD: 940/217,852); and 218,507 participants (cases/controls for UC: 2,701/215,806) (34). Since 2017, FinnGen has been a large-scale national effort that aims to improve human medicine by gathering genetic data and health record information from Finnish health registries and Biobanks, respectively. The detailed information on all traits involved was summarized in **Table 1**. Since all of the data are GWAS summary statistics that are available to the public, no further ethical approval or informed permission was needed.

**Table 1:** Detail of the data for the cohort population.

Trait	Gwas ID	Data source	Sample size	Case/control	Number of SNPs	Population	Year
IBD	finn-b-K11_IBD	FinnGen	218792	5,673/213,119	16,380,466	European	2021
CD	finn-b-K11_KELACROHN	FinnGen	218792	940/217,852	16,380,466	European	2021
UC	finn-b-K11_UC_STRICT	FinnGen	218507	2,701/215,806	16,380,466	European	2021
PLT	ebi-a-GCST90002402	UK Biobank	408,112	/	40,299,783	European	2020
MPV	ebi-a-GCST90002395	UK Biobank	408,112	/	40,299,375	European	2020
PDW	ebi-a-GCST90002401	UK Biobank	408,112	/	40,300,122	European	2020
PCT	ebi-a-GCST90002400	UK Biobank	408,112	/	40,299,196	European	2020

### Selection of instrumental variables

IVs were chosen as independent SNPs at genomewide significance (P<5×10^−8^) for every exposure taken into account in univariable MR analysis. Pairwise linkage disequilibrium, or independent SNPs, were found using criteria of (r^2^<0.001, clumping window=10,000 kb). To find and eliminate outlier instruments, MR pleiotropy residual sum and outlier (MR-PRESSO) analyses were carried out. The cumulative strength of the chosen SNPs was assessed using the F-statistic (F = beta^2^/se^2^), where beta denoted the exposure’s effect value and se denoted the exposure’s standard error. This helped to prevent weak instrument bias. F>10 is required to access the whole SNPs collection (35). The F-statistics used in the univariable MR analyses are provided in **Supplementary Table 1**.

### Statistical analysis

Reverse causation can lead to an incorrect inference that the exposure and the outcome are causally connected if variations in the outcomes that exhibit greater relationships with outcomes than with exposures are employed in the MR analyses (36). Consequently, we must exclude the SNPs that have an outcome of P<5×10^−8^. And then, prior to analysis, we first harmonized exposure and outcome data to make alignments on effect alleles to the forward strand, if it is specified or could be inferred based on the allele frequency. Ambiguous SNPs with non-concordant alleles and palindromic SNPs that may create uncertainty regarding the identification of the effect allele in the exposure and outcome GWASs were excluded for further MR analyses (37, 38). After identifying the IV sets using the aforementioned selection criteria, we estimated the total effects using MR analysis. We performed a significance analysis using the IVW approach. Assuming that all SNPs are legitimate instrumental factors, this technique yields the maximum power estimate. When all IVs are genuine and horizontal pleiotropy is balanced, this method yields unbiased estimates of causal links even in the presence of variability across SNPs (39). The MR-Egger regression was used in secondary analyses to account for pleiotropy and assess the findings’ robustness. Although its power is limited, the MR-Egger method can identify and rectify directional pleiotropy. Even in the event that the second and third assumptions are false, it accounts for the directed pleiotropic effects of genetic instruments (40). The MR-Egger test produces a consistent causal estimate and a valid test of the null causal hypothesis, even in the case when all genetic variations are invalid (40). Nevertheless, MR-Egger shows poor statistical accuracy and is vulnerable to outlying genetic variations (41). The weighted median approach is the third method. It is substantially and continuously more accurate than the MR Egger approach and more resilient to violations of causal effects (42). It is predicated on the supposition that more than half of the IVs are believable. Furthermore, outliers and high-leverage genetic variants won’t have an impact on it (42). Otherwise, the IVW outcomes took precedence. The OR and accompanying 95% CI on the outcome risk of corresponding unit changes in exposure were used to represent the MR results. To evaluate the relative risk brought on by the existence of the illness of interest, the OR and 95% CI were shown. P<0.05 was used to indicate statistical significance in the univariable MR analysis for the findings of sensitivity analyses on the causal effects of exposures and outcomes. To depict the MR data, scatterplots, forest plots, and funnel plots were created in the interim.

We also assessed horizontal pleiotropy for significant estimates using the intercept tests of MR-Egger regression and MR-PRESSO. MR-Egger regression yielded an intercept, and intercept values that differ from zero indicate pleiotropy (here assessed using a p-value <0.05), which was suggestive of an overall directional pleiotropy (43). Using the global and SNP-specific observed residual sum of squares, the MR-PRESSO method screened for general horizontal pleiotropy (global test) and outliers (outlier test), with a significant threshold of 0.05 (44). Additionally, after eliminating outliers, it provided causal estimates and contrasted the raw values with the distortion. Additionally, 10,000 distribution points were allocated. By gradually eliminating each IV, leave-one-out analysis was performed in order to identify bias caused by a heterogeneous variation. In order to identify heterogeneity (p<0.05 shows heterogeneity), we also calculated the Cochrane’s Q value, which allowed us to identify the existence of pleiotropy (45). Each SNP’s heterogeneity in terms of causative effects was assessed using Cochran’s Q value (46). For the second and third assumptions to be satisfied, horizontal pleiotropy must be assessed (38). R statistical program (version 4.3.1, R Foundation for Statistical Computing, Vienna, Austria, 2023; https://www.R-project.org) was used for all statistical analyses, together with the Two-Sample MR and MR-PRESSO Packages (38).

## Results

### Selection of instrumental variables

Altogether, 477 index SNPs were shown to be possible genetic IVs for IBD, 482 SNPs for CD, and 479 SNPs for UC when PLT was taken into account as an exposure factor. In the presence of MPV as an exposure factor, 453 index SNPs were shown to be putative genetic IVs for IBD, 455 SNPs for CD, and 454 SNPs for UC, in that order. PDW as an exposure factor led to the identification of 379 index SNPs as putative genetic IVs for IBD, 378 SNPs for CD, and 375 SNPs for UC, in that order. In the case of PCT as an exposure factor, possible genetic IVs for IBD, CD, and UC were found to be 452 index SNPs, 454 SNPs, and 453 SNPs, respectively. Not only have all of these SNPs been harmonized and palindromic SNPs with intermediate allele frequencies removed, but they have also undergone the MR-PRESSO test, which was run in order to identify and eliminate outlier IVs. Once the outlier IVs were eliminated, MR estimations were reexamined. Thus, the SNPs listed above were taken into account for the MR analysis. Furthermore, each SNP’s F-value was greater than 10, which suggests that there is a minimal possibility of weak instrumental variable bias.

### Mendelian randomization analysis

**Figure 3:**
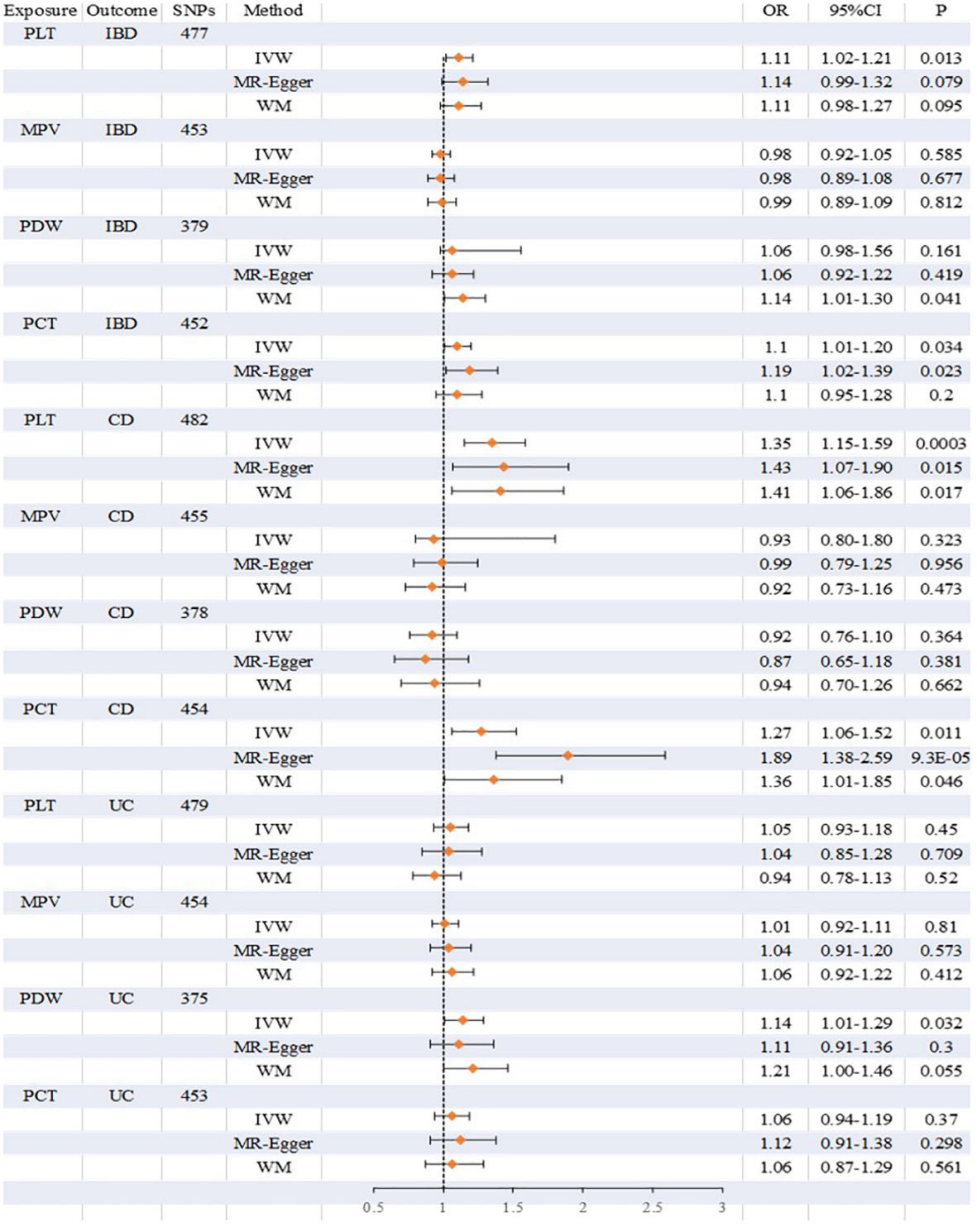
Detailed results on the association between platelet indices (PLT, MPV, PDW, PCT) and IBD, CD and UC.

**Figure 4:**
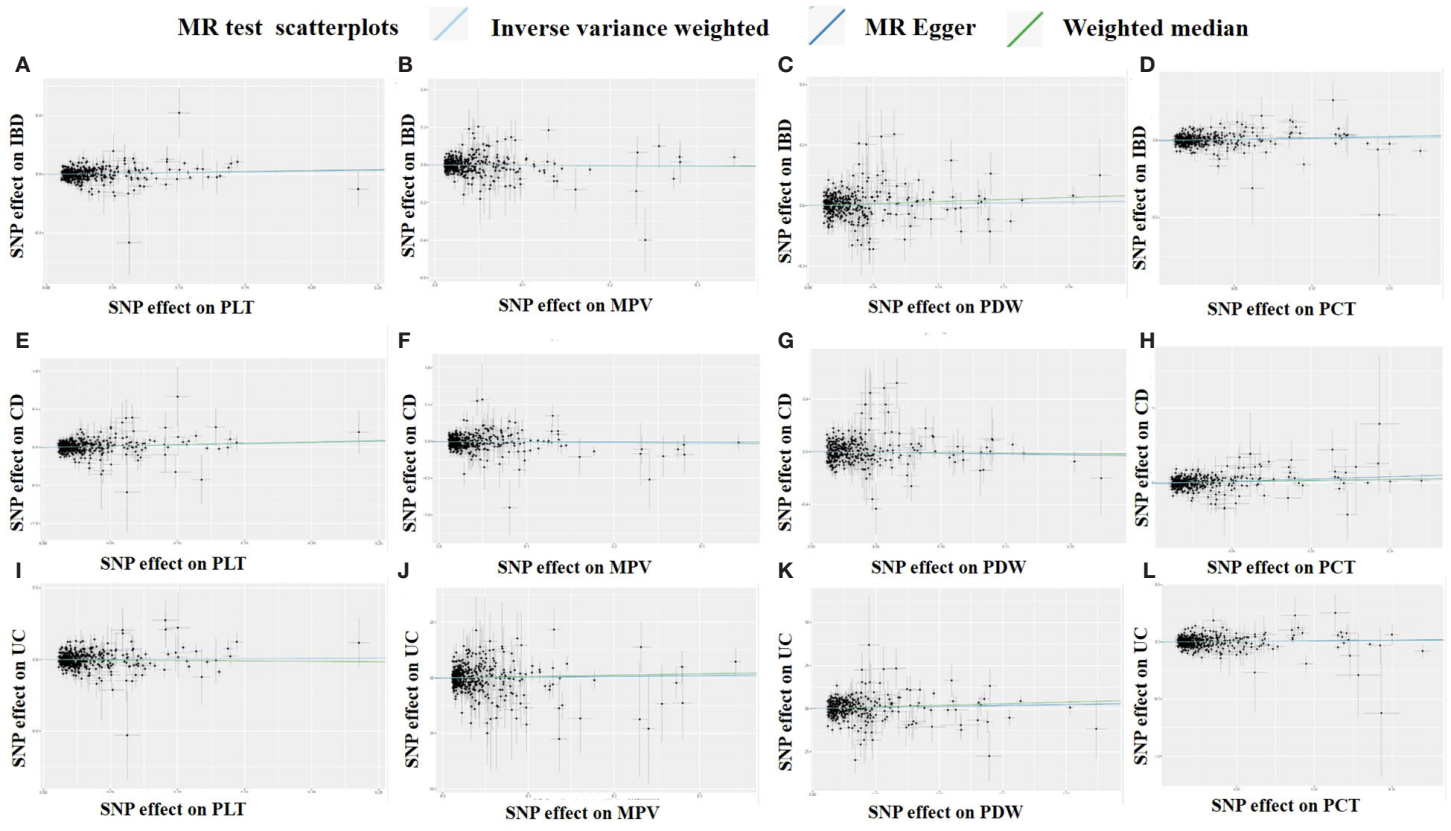
Scatter plots of the MR analysis. **(A)** PLT on IBD. **(B)** MPV on IBD. **(C)** PDW on IBD. **(D)** PCT on IBD. **(E)** PLT on CD. **(F)** MPV on CD. **(G)** PDW on CD. **(H)** PCT on CD. **(I)** PLT on UC. **(J)** MPV on UC. **(K)** PDW on UC. **(L)** PCT on UC.

Overall, there was inconsistency in the results from the three approaches used to establish a causal relationship between platelet indicators (PLT, MPV, PDW, and PCT) and IBD (UC and CD). According to the IVW method’s MR estimations, there is a significant correlation between PLT and IBD (OR:1.11, 95%CI:1.02 to 1.21, P:0.013). However, IBD was not associated with the findings of the MR-Egger or weighted median techniques (OR:1.14, 1.11,95%CI:0.99 to 1.32,0.98 to 1.27, P:0.079,0.095), respectively. Likewise, there is a close link between PCT and IBD. IVW produced the following results: OR:1.10, 95%CI:1.01 to 1.20, P:0.034. OR:1.19, 95%CI:1.02 to 1.39, P:0.023 was the MR-Egger. However, there was no significant difference using the weighted median approach (OR:1.10, 95%CI:0.95 to 1.28, P:0.2). PLT and PCT were related to CD, whereas PDW was connected to UC, according to further study of the two subtypes. IVW (OR:1.35, 95%CI:1.15 to 1.59, P:0.0003), MR-Egger (OR:1.43, 95%CI:1.07 to 1.90, P:0.015), and weighted median (OR:1.41, 95%CI:1.06 to 1.86, P:0.017) were the values obtained from PLT to CD. PLT and CD have strong relationships, according to the findings of all three approaches. A comparison between PCT and CD revealed similarities in the IVW (OR:1.27, 95%CI:1.06 to 1.52, P:0.011), MR-Egger (OR:1.89, 95%CI:1.38 to 2.59, P:9.3×10^-5^), and weighted median (OR:1.36, 95%CI:1.01 to 1.85, P:0.046). PCT was closely associated to CD, according to the findings of all three methodologies. However, the only elevated factor with regard to UC was PDW. And only IVW’s finding (OR:1.14, 95%CI:1.01 to 1.29, P:0.032) was remarkable. We found no relationship between other platelet indices and IBD, CD, and UC; the detailed results and scatterplots are listed in **Figures 3**, **4**. And the forest plots and funnel plots are shown in **Supplementary Figures 1**, **2**.

### Sensitivity analysis

While some of the Cochran Q test findings showed heterogeneity, the major outcome of the random effects IVW analysis allowed for some heterogeneity. All except one of the p-values for the MR-Egger intercept were greater than 0.05. The results and details are provided in **Supplementary Table 2**. Furthermore, our results’ robustness was further validated by the fact that leave-one-out analysis failed to find any outlier IVs (**Supplementary Figure 3**). Additionally, following the global MR-PRESSO testing, we had to exclude a few SNPs. However, there were all significant SNPs after removing the outliers. The MR-PRESSO distortion test results showed the causal effect of genetically predicted platelet indices on IBD (CD, UC) after correction by removing outliers. On the other hand, genetically predicted platelet indices were shown to raise the risk of IBD (CD, UC) in both corrected and uncorrected data (**Table 2**).

**Table 2:** The MR-PRESSO test’s results.

Exposure	Outcome	Raw			Outlier corrected			Global P	Number of outliers	Distortion P
		OR	95%CI	P	OR	95%CI	P			
PLT	IBD	1.10	1.01–1.20	0.031	1.11	1.02–1.21	0.009	<1e-04	5	0.787
MPV		0.99	0.92–1.05	0.640	0.98	0.92–1.05	0.575	<1e-04	2	0.947
PDW		1.06	0.98–1.56	0.216	-	-	-	<1e-04	NA	NA
PCT		1.10	1.00–1.20	0.116	1.10	1.01–1.20	0.058	<1e-04	1	0.822
PLT	CD	1.35	1.15–1.59	0.0003	-	-	-	0.502	NA	NA
MPV		0.93	0.80–1.80	0.324	-	-	-	7e-04	NA	NA
PDW		0.92	0.76–1.11	0.371	0.92	0.76–1.10	0.455	0.006	1	0.810
PCT		1.27	1.06–1.52	0.011	1.27	1.06–1.52	0.008	0.031	1	0.945
PLT	UC	1.05	0.93–1.18	0.432	1.05	0.93–1.18	0.436	<1e-04	3	0.978
MPV		1.01	0.92–1.11	0.829	1.01	0.92–1.11	0.699	<1e-04	1	0.908
PDW		1.14	1.01–1.29	0.032	1.14	1.01–1.29	0.016	<1e-04	4	0.910
PCT		1.06	0.94–1.19	0.374	1.06	0.94–1.19	0.430	<1e-04	1	0.909

## Discussion

This is the first MR research that we are aware of that examines the relationship between platelet indices and IBD (UC and CD). The purpose of the current study was to investigate the relationship between IVs of the four platelet indices and IBD (CD, UC). We discovered in the univariable MR that a rise in IBD and CD was correlated with the amounts of PLT and PCT predicted by the provided genetics, while PDW was linked to UC. But there was no significant correlation between other platelet indicators and IBD (CD, UC). According to these results, PLT and PCT are the essential characteristics that generate favorable correlations between IBD and CD. PDW may only relevant to UC.

A two-sample MR analysis of the relationship between platelet indices and IBD was conducted for this investigation. There was shown to be a strong relationship between platelet indices and IBD. In order to better understand the association between platelet indices and IBD and to develop therapies for the disease, a greater study of the correlations between various platelet indices and IBD utilizing bigger and more diverse data sources is necessary. However, although they are categorized as IBD, CD, and UC, they are not the same in terms of pathophysiology, symptoms, complications, natural courses, and sequelae. In addition to severely impairing a patient’s quality of life, CD and UC both increase mortality and financial burden (12, 14, 47). Although the exact cause of IBD (CD, UC) is still unclear, genetic vulnerability, environmental factors, and the gut microbiome may all be significant (48). Further evidence of these two distinct situations was found in our research.

As is well known, PLT counts the number of platelets per unit volume of blood, PCT represents the proportion of blood volume occupied by platelets, and MPV indicates the average size of platelets. As a result, PCT is connected with the products of MPV and PLT, and may be thought of as a sort of analog of the total platelet volume. PDW, in comparison to PLT, PCT and MPV, is another significant metric. Thus, elevated indices may suggest that platelets play a part in understanding the IBD process (CD, UC). In our study, we have found there is a relevance between PLT, PDW, and PCT with IBD (CD, UC), so the platelet indices reflect this phenomenon and may be useful indicators for assessing the course of IBD (CD, UC). In clinical practice, it is important to highlight the independent and prominent roles that PLT, PCT, and PDW play among the four platelet indices.

Excessive clotting or unusual bleeding are the outcomes of elevated platelet levels (49). Because of the close involvement of their membrane receptors at different stages of the blood-coagulation cascade (50), a sequence of biochemical reactions that take place in the body in response to injury or damage to blood vessels, platelets play a critical role as the defenders of the integrity of the blood vasculature. The exterior membrane of platelets is extremely active and functional, expressing different integrin, glycoproteins, and antigens (1). These membrane constituents play a crucial role in coordinating the intricate interplay between platelets and sub endothelial structures that are exposed due to blood vessel wall damage. Additionally, proteins that make up fibrin clots and plasma coagulation factors and activators interact with biomolecules produced on platelet membranes. Membrane glycoproteins identify blood clotting factors and play a key role in platelet adherence and activation. Platelet membranes strongly express GPIIb/IIIa, GPIb-IX-V, GPVI, and P2Y12, all of which are essential in the hemostatic process that comes before the wound-healing phase (51). The immunological response of the body is improved by platelets. It has been demonstrated that platelet-derived CD40L may stimulate monocyte differentiation into dendritic cells (DC), DC maturation, and co-stimulatory molecule upregulation (52). This role of platelet-derived CD40L may be particularly important for autoimmune illnesses like systemic lupus erythematosus, where platelets stimulate B-cell secretion of antibodies via inducing DC differentiation and type-I interferon release (53). But IBD is an autoimmune disease that recurs frequently, causing intestinal bleeding, inflammatory responses, and EIMs such as cardiovascular problems. Furthermore, the precise aspects of its pathophysiology are yet unknown, but they appear to be linked to immune response problems and genetic predisposition. So combining the function of platelets and the MR results we obtained, platelet-related indices are indeed closely related to IBD and predict its occurrence and development.

We discovered the link between platelet indices and IBD (UC and CD), as previously mentioned. However, three presumptions relevance, independence, and exclusion-restriction are necessary for IVs to be valid in MR. The second and third assumptions, however, are dependent on every potential confounding factor of the exposure-outcome connection, both measurable and unmeasured, and only the first can be completely empirically evaluated. To provide a consistent estimate of the causative effect, all genetic variations included in the research as IVs must meet the MR assumptions for the IVW method (42). Both the weighted median and the MR-Egger methods were used to verify this. Even in cases where all genetic effects are null due to violations of the third assumption mentioned above, the MR-Egger approach reliably predicts the genuine causal impact under a lesser assumption (54). However, if all genetic variants have a comparable degree of connection with the exposure, then MR-Egger regression estimates become less accurate. On the other hand, if no single genetic variation accounts for more than 50% of the weight, the weighted median approach will yield a consistent estimate only if at least 50% of the weight originates from legitimate genetic variants. When it comes to faulty genetic variations, the weighted median method permits a more widespread violation of the MR assumptions than the MR-Egger method does (42). Therefore, we think that the remaining results suggest a causal relationship between platelet indices and IBD, even if an MR-Egger technique observation yielded a non-significant estimate.

Although we have identified a relationship between platelet indices and IBD through the MR study. There were a few more restrictions on this study. Firstly, it is probable that the putative gender-specific effects on the relationship were overlooked since we did not separate platelet indices and IBD (UC and CD) by gender. The UK Biobank sample was used for the GWAS of characteristics linked to platelet indices, while FinnGen provided data on IBD (CD, UC). As a result, bias and sample overlap are possible in relation to this fact (55). Furthermore, even though steps have been taken to identify and eliminate outlier SNPs, we cannot totally rule out the possibility that heterogeneity will have an impact on the results. Moreover, our work has demonstrated a causal association between platelet indices and IBD (UC and CD); nevertheless, additional research is necessary as the specific underlying processes are still unclear. Then, even with an MR research design, confounding cannot be totally minimized because the risk factors for IBD (CD, UC) comprise not just genetic variables but also other factors, such as environmental ones. Finally, the study only contained four platelet indices; more hematological indicators associated with platelets may exist, meaning that the relative importance of PLT, PCT, and PDW may need to be adjusted when considering other features.

## Conclusions

Evidence supporting PLT, PCT, and PDW as distinct and predominant features explaining the relationship to IBD (CD, UC) may be found in the current MR investigation. Comprehending the function of platelets and their associated characteristics is beneficial for both public and clinical health. To strengthen the case for antiplatelet medication as the main preventive measure in IBD patients, stratified randomized controlled trials are also required. Our MR investigation showed that PLT and PCT had a connection to IBD and CD meanwhile that PDW had a relation to UC. To a certain extent, platelets and their associated characteristics influence the development of IBD (UC, CD). A possible preventative method for IBD might involve focusing on these characteristics. Further research is required to determine the precise mechanism and validate the therapeutic benefits of this kind of preventive therapy.

## Data availability statement

The datasets presented in this study can be found in online repositories. The names of the repository/repositories and accession number(s) can be found in the article/**Supplementary Material**.

## Ethics statement

Ethical approval was not required for the study involving humans in accordance with the local legislation and institutional requirements. Written informed consent to participate in this study was not required from the participants or the participants’ legal guardians/next of kin in accordance with the national legislation and the institutional requirements. Written informed consent was not obtained from the individual(s) for the publication of any potentially identifiable images or data included in this article because online public data does not require informed consent.

## Author contributions

HL: Data curation, Formal analysis, Investigation, Software, Writing – original draft, Writing – review & editing. TL: Conceptualization, Funding acquisition, Writing – review & editing.
