# Supplementary material for: Platelet indices and inflammatory bowel disease: a Mendelian randomization study
Source: Front Immunol. 2024 Jul 9;15:1377915. doi: 10.3389/fimmu.2024.1377915 (PMC11263279; doi:10.3389/fimmu.2024.1377915)

Supplementary Material

Supplementary Table 2

Sensitivity Analysis through Cochran Q test and MR-Egger intercept test

Exposure	Outcome	SNPs	Het		Plei	
			Q	P	Intercept	P
PLT	IBD	477	678.1873	2.87E-09	-0.0010	0.673
MPV		453	588.2722	1.56E-05	0.0002	0.941
PDW		379	532.8796	2.44E-07	0.0002	0.952
PCT		452	643.6105	6.06E-09	-0.0030	0.196
PLT	CD	482	480.2645	0.501	-0.0022	0.633
MPV		455	552.4716	0.001	-0.0038	0.419
PDW		378	449.5625	0.006	0.0022	0.683
PCT		454	511.9186	0.029	-0.0147	0.003
PLT	UC	479	670.5051	1.31E-08	0.0002	0.947
MPV		454	571.1968	0.0001	-0.0016	0.589
PDW		375	532.7413	1.21E-07	0.0012	0.731
PCT		453	615.8007	4.26E-07	-0.0021	0.519

Supplementary Figure 1

Forest plots of the MR analysis.

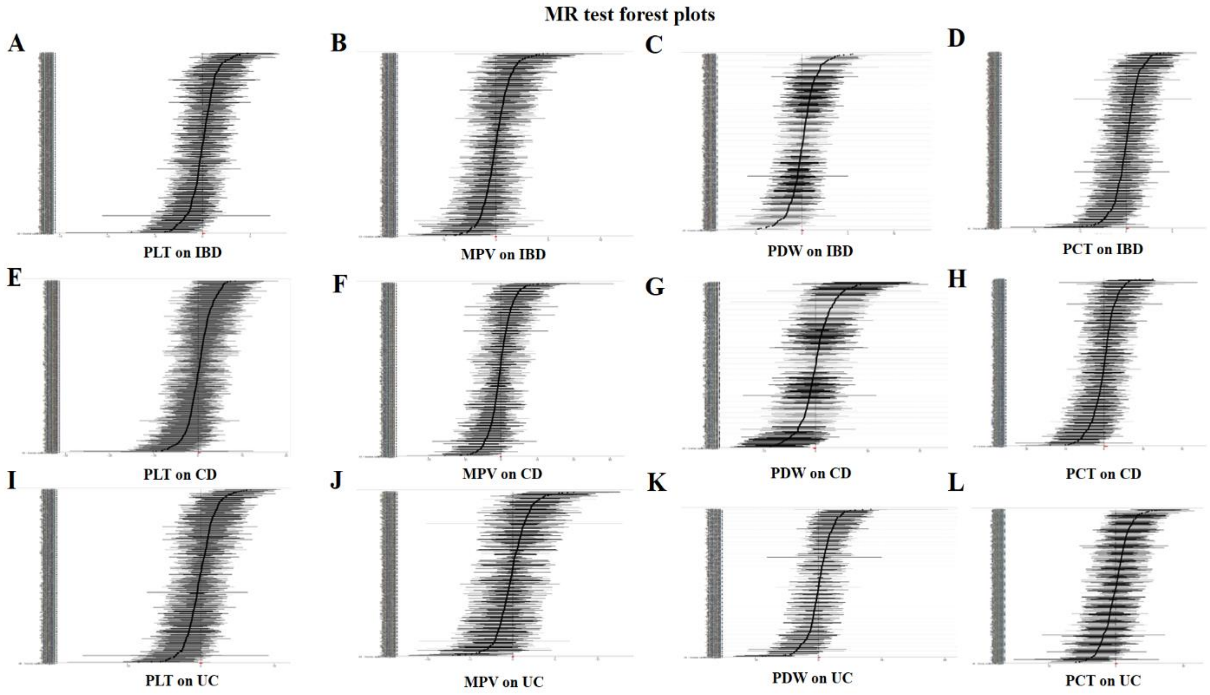

Supplementary Figure 2

Funnel plots of the MR analysis.

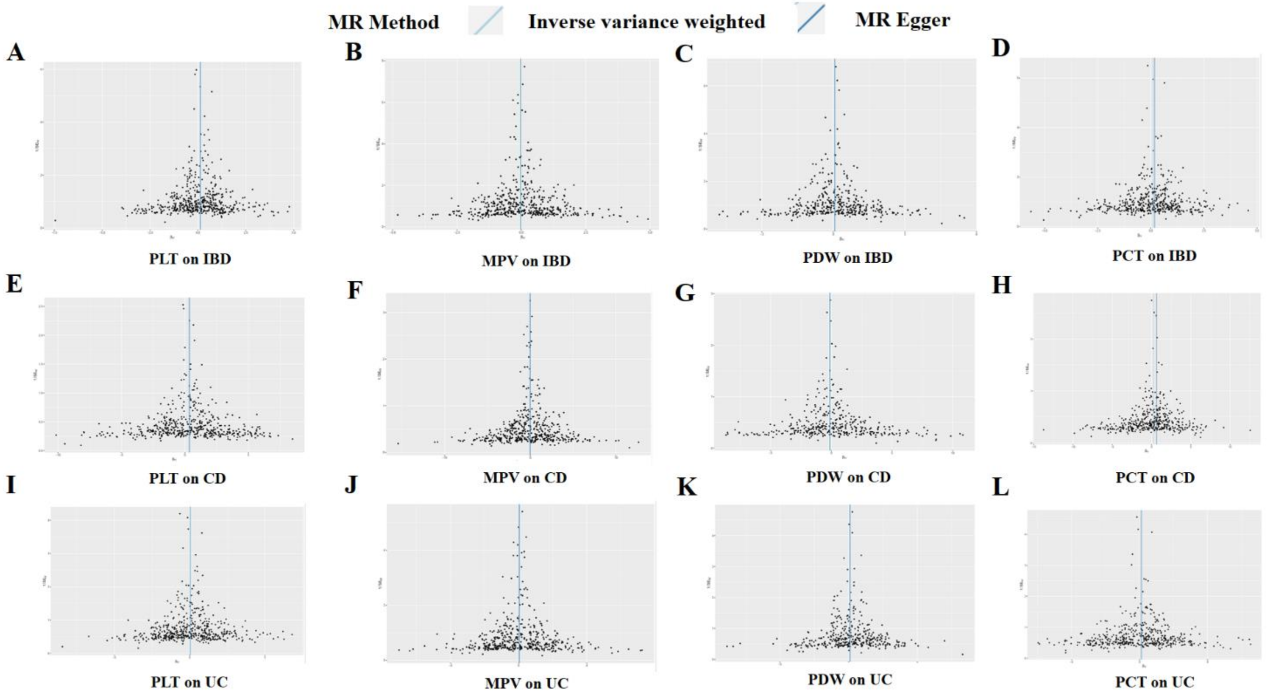

Supplementary Figure 3

Plots of the leave-one-out analysis.

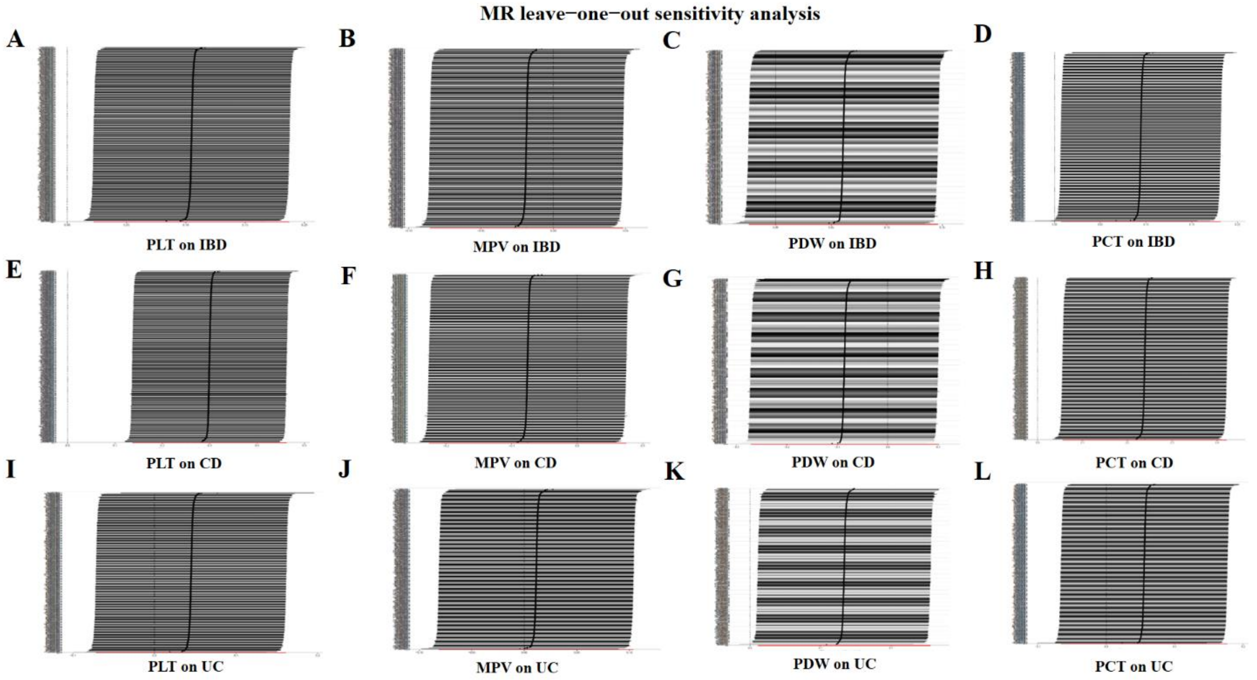

Supplement: Supplementary file 2 [file Table_2.pdf]
